# The iceberg model of suicidal ideation and behaviour in Danish adolescents: integration of national registry and self-reported data within a national birth cohort

**DOI:** 10.1007/s00787-024-02503-w

**Published:** 2024-06-25

**Authors:** Stine Danielsen, Katrine Strandberg-Larsen, Keith Hawton, Merete Nordentoft, Annette Erlangsen, Trine Madsen

**Affiliations:** 1 Section of Epidemiology, Faculty of Health and Medical Sciences, University of Copenhagen, Øster Farimagsgade 5, bd. 24, PO Box 2099, Copenhagen, DK - 1014 Denmark; 2 Centre for Suicide Research, Department of Psychiatry, University of OxfordWarneford Hospital, Oxford, OX3 7JX England; 3 Danish Research Institute for Suicide Prevention – DRISP, Mental Health Center Copenhagen, University of Copenhagen, Gentofte Hospitalsvej 15, Opg. 15, 4. floor, Hellerup, DK – 2900 Denmark; 4 Department of Clinical Medicine, Faculty of Health and Medical Sciences, University of Copenhagen, København, Denmark; 5 Centre for Mental Health Research, Research School of Population Health, The Australian National University, Canberra, Australia; 6 Department of Mental Health, Johns Hopkins Bloomberg School of Public Health, Baltimore, MD USA

**Keywords:** Suicidal behaviour, Self-harm, Suicidal ideation, Adolescence

## Abstract

Hospital records are used to identify suicide attempts in many countries but not all individuals present to hospital after a suicide attempt i.e., suggesting a ‘hidden number’. Our aim was to present the prevalence of suicide ideation, plans, attempts, and suicides among Danish adolescents, including attempts not resulting in hospital contact. The study population consisted of participants in the Danish National Birth Cohort participating in an 18-year follow-up, with individual-level linkage to national register data. Prevalence was estimated with a variable with mutually exclusive categories ranging from no suicidality to self-reported suicide ideation, -plans, -attempt and hospital-recorded suicide attempt and stratified on sex and parental income. The ‘hidden number’ was estimated as the ratio between suicide attempts with and without hospital contact. Among 47 858 participants, all aged 18-years, 36% girls and 28% boys reported suicide ideation at least once in their life. In addition, 6% girls and 3% boys had either reported or been recorded with a suicide attempt. For every attempt recorded in the hospital setting, two girls (ratio, 1:2) and six boys (ratio, 1:6) reported having attempted suicide without hospital contact. The prevalence of any suicide attempt was 8% and 3% in the lowest and highest income group, respectively. Before age 18, 0·011% girls and 0·016% boys had died by suicide. In conclusion, suicidal ideation and behaviour are common in adolescents and there is a substantial ‘hidden number’ of adolescents with suicide attempt. These results emphasize the need for early age suicide preventive interventions in community-settings e.g., school environments.

**Supplementary Information:**

The online version contains supplementary material available at 10.1007/s00787-024-02503-w.

## Introduction

Suicidality is a public health concern and since the millennium, increasing rates of hospital-recorded suicide attempts have been reported among adolescents in Denmark, especially among girls [1]. Although suicides are rare among adolescents, it is one of the leading causes of death in this age group [2, 3]. Suicidal thoughts and behaviour are indicators of poor mental health and suicide attempt is one of the strongest risk factors for later death by suicide [4]. People who present to hospital with a suicide attempt or die by suicide represent overt manifestations of suicidal behaviour. These people are the visible tip of the iceberg of suicidal behaviour, while other suicidal events in the community might be hidden; thus, suggesting a ‘hidden number’ [2, 5–7]. 

Findings based on self-reported data from large community samples and a meta-analysis suggest that 12–22% of young adults aged 13–22 years have experienced suicidal ideations and 2–7% have attempted suicide, with both being more common among girls [8–11]. Few studies have been able to simultaneously study self-reported suicidality and hospital records of suicide attempt, as well as death by suicide [5, 6]. Findings from such studies suggest that girls have higher rates of both hospital-recorded and self-reported self-harm, including suicide attempt, while boys have higher rates of suicide [5, 6]. A 1:7–10 and 1:7–20 ratio between hospital presentations for suicide attempt and self-reported suicide attempt was found among boys and girls, respectively. However, in these studies, individual-level linkage between data sources did not occur. Individual-level linkage of self-reported and hospital-recorded suicidality allows estimation of how many in a general population sample have had suicide ideation, attempted suicide and how many of these individuals present to hospital with suicide attempts.

The primary aim of this study was to estimate the prevalence of suicide ideation, plans, attempts, and suicides among Danish adolescents by individual-level linkage of self-reported and register-based data and to estimate the ‘hidden number’ of adolescents with suicide attempts. We have also examined differences related to sex and parental income.

In this study, as well as in most research on suicide attempts in Denmark, we use an algorithm developed to identify suicide attempts in Danish registers [1]. The ability of this algorithm to accurately identify a suicide attempt, especially among adolescents, has been questioned [12, 13]. Our second aim was to evaluate the validity of the existing algorithm for identification of suicide attempts by comparison to self-reported data on suicidality.

## Method

### Design and study population

The study population was based on the Danish National Birth Cohort (DNBC), a cohort including children born in Denmark from 1996 to 2003 [14]. Approximately 100 000 pregnant women were recruited to the cohort by their general practitioner during the first antenatal care visit and gave birth to 96 822 liveborn children. The children were followed from prenatal life to the beginning of adulthood through multiple data collections. In 2016–2021, participants at age 18 years and three months were invited to participate in an online questionnaire survey (DNBC-18). Out of 89 205 eligible participants, 47 858 (54%; girls: 63%; boys: 44%) responded to the questionnaire including all items concerning suicidality (Supplementary Fig. 1). These participants constituted the study population except for suicide. Information on suicide plans became available halfway through the data collection, and the sub-group of participants (*N* = 19 186) who completed the DNBC-18 after May 2019 constituted a sub-population. The DNBC is a nested sub-set of the entire Danish population, and we defined the background population for the DNBC as liveborn children born during the recruitment years (*N* = 451 768). All individuals living in Denmark are issued with a unique personal ID number, which allows for individual-level linkage of various administrative data registers. Thus, it was possible to link data from the DNBC with data on health-related and sociodemographic information from nationwide registers (Supplementary Table 1).

### Outcome measures

Data on suicide deaths were identified in the Cause of Death Register as a code of X60–X84 in the International Classification of Diseases, version 10 (ICD-10) or where the manner of death was listed as suicide [15]. 

Hospital records of suicide attempts were identified with an algorithm and retrieved from the National Patient Register using diagnoses classified according to ICD-10 (Table 1) [16]. This algorithm has previously been applied and referred to as ‘probable suicide attempt’ [1, 12, 17]. The algorithm defined hospital-recorded suicide attempts by (a) ICD-10 codes X60-X84 or where suicide attempt was recorded as the reason for contact. As suicide attempt by definition (a) is under-recorded in Danish hospital settings, the algorithm also includes contacts likely to represent suicide attempt defined by contacts with (b) intoxication with specific drugs, (c) a psychiatric diagnosis in combination with intoxication with all drugs and biological substances except alcohol or d) a psychiatric diagnosis in combination with injuries to the lower forearm (Table 1) [1, 12, 13, 17]. Only hospital-recorded suicide attempts, which occurred prior to the date of completion of the DNBC-18 questionnaire, were included.

**Table 1:** Definitions of register-based and self-reported suicidality

Classifications of register-based suicidality		
Suicide	Cause of death classified according to the International Classification of Diseases, version 10 (ICD-10) as X60-X84 or Y87.0.	
Hospital-recorded suicide attempt	a) All hospital contacts with a main or supplementary ICD-10 diagnosis of X60-X84 or where reason of contact code for “suicide attempt”. b) Main diagnosis accidental intoxication with weak analgesic drugs, antiepileptics, sleeping pills, antiparkinsonian drugs, psychotropics, and carbon monoxide (ICD-10: T39, T40 except T40.1, T42, T43, T58). c) Main diagnosis of psychiatric disorder diagnosis (ICD-10: F00-F99) in combination with a sub-diagnosis of intoxication with drugs and biological substances (ICD-10: T36-T50, T52-T60). d) Main diagnosis of psychiatric disorder diagnosis (ICD-10: F00-F99) in combination with a sub-diagnosis of injuries to the lower forearm (ICD-10: S51, S55, S59, S61, S65, S69).	
**Questions on self-reported suicidality in the Danish National Birth Cohort 18-year follow-up** ^1^		**Reply options**
Suicide attempt	Have you ever tried to take your own life?	Yes, No or Do not know
Suicide plans^2^	Have you ever had suicide plans (considered methods, done preparations)?	Yes, No or Do not know
Suicide ideation	Have you ever thought about taking your own life (even though you would not do it)?	Yes, No or Do not know

Given that > 80% suicide attempts recorded before age 10 years were among children aged 0–3 years and most likely due to accidents, we opted only to include episodes recorded from age 10 years or older.

Participants in DNBC-18 were asked whether they ever have had suicide ideation, -plans and -attempts (Table 1). If the answer was affirmative, they would subsequently receive a question regarding whether this had been within the last year. Participants could answer ‘yes’, ‘no’ and ‘do not know’ to these questions. We categorised ‘do not know’ as ‘no’. The DNBC-18 was pilot tested by young people.

Register-based and self-reported measures of life-time suicidality were combined into a hierarchical variable with following mutually exclusive categories: *none, self-reported suicide ideation, self-reported suicide plans, self-reported suicide attempt, hospital presentation for suicide attempt*. Each individual was categorised according to their highest level of suicidality. Thus, adolescents with both self-reported and hospital-recorded suicide attempt would be categorised solely as hospital-recorded suicide attempt and only adolescents without a hospital record of suicide attempt were categorised as self-reported suicide attempt. Information of suicide plans was only available for a subset of participants. As death precluded participation in the DNBC-18, the prevalence of suicide was calculated for all Danes born during the same period as DNBC children. Suicide presented as the highest level of suicidality.

### Statistical analyses

Sample weights were calculated and applied to adjust for differential participation and attrition in DNBC [14, 18, 19]. Based on characteristics of the background population i.e., all Danes born during the same period as DNBC children, larger weights were used for individuals with a lower probability of participating in DNBC-18, while lower weights were assigned to groups who were over-represented [20]. Weights were calculated separately for boys and girls, and for each parental income quartile and truncated to the cut-off value median + 5*IQR [21]. The probability of having participated in DNBC-18 was estimated in a logistic regression model with the following variables: sex (when not stratified upon), parental income (when not stratified upon), highest parental education, parental job-status, maternal age, parity, co-living parents, out-of-home placement, any childhood or adolescent psychiatric diagnosis, and any history of parental psychiatric diagnosis (Table 2, Supplementary Table 1). All, except sex, maternal age and parity were retrieved at 18 years of age. Parents were defined as the parents registered on the birth certificate. Unweighted estimates are provided in the supplement (Supplementary Tables 2–5).

**Table 2:** Characteristics of the background population (all Danes born during the same period as DNBC children) and unweighted and weighted participants in the 18-year follow-up of the Danish National Birth Cohort (DNBC-18)

	Background population (*N* = 449 288)	Unweighted DNBC-18 (*N* = 47 858)	Weighted DNBC-18
Characteristics	*N* (%)	*N* (%)	(%)
**Sex** ^1^			
Girls	218 869 (49)	27 727 (58)	(50)
Boys	230 419 (51)	20 131 (42)	(50)
**Parental income** ^**2**^			
Q1 (lowest)	110 272 (25)	5959 (12)	(23)
Q2 (second lowest)	110 596 (25)	11 358 (24)	(26)
Q3 (second highest)	110 690 (25)	14 292 (30)	(26)
Q4 (highest)	110 807 (25)	16 217 (34)	(26)
Missing	6923 (1)	32 (< 0.5)	(< 0·5)
**Highest parental education** ^**2**^			
Elementary school	32 048 (7)	1083 (2)	(6)
Vocational education	144 168 (32)	12 418 (26)	(34)
High School	25 395 (6)	2262 (5)	(6)
Higher education	206 918 (46)	30 396 (64)	(47)
Missing	40 759 (9)	1699 (4)	(7)
**Parental job-status** ^**2**^			
In job	326 218 (73)	40 674 (85)	(75)
Not in job	109 814 (24)	6718 (14)	(23)
Absent leave	6211 (1)	427 (1)	(1)
Missing	7045 (2)	(< 0·5)	(< 0·5)
**Maternal age at birth** ^**1**^			
≤25	91 701(20)	6354 (13)	(19)
26–30	175 562 (39)	20 410 (43)	(39)
31–36	149 009 (33)	17 656 (37)	(34)
>36	32 857 (7)	3424 (7)	(8)
Missing	158 (< 0·5)	14 (< 0·5)	(< 0·5)
**Parity (number of liveborn children at birth of index child)** ^**1**^			
1	189 332 (42)	22 271 (47)	(43)
2	162 651 (36)	16 623 (35)	(37)
≥3	84 571 (19)	7265 (15)	(17)
Missing	12 734 (3)	1699 (4)	(3)
**Co-living parents** ^**2**^			
No	200 326 (45)	18 087 (38)	(46)
Yes	236 926 (53)	29 738 (62)	(54)
Missing	12 036 (3)	33 (< 0·5)	(< 0·5)
**Out-of-home placement** ^**3**^			
No	434 557 (97)	47 266 (99)	(97)
Yes	14 731 (3)	592 (1)	(3)
**Psychiatric diagnosis in adolescent** ^**3**^			
No	394 493 (88)	42 753 (89)	(87)
Yes	54 795 (12)	5105 (11)	(13)
**Parental psychiatric diagnosis** ^**3**^			
No	360 055 (80)	41 064 (86)	(80)
Yes	89 233 (20)	6794 (14)	(20)
**Self-reported data on suicidality**			
**Suicide ideation**			
No	-	28 843 (60)	27 066 (59)
Yes	-	16 828 (35)	16 655 (36)
Do not know	-	2187 (5)	2040 (4)
**Suicide plans** ^**4**^			
No	-	16 811 (88)	15 719 (86)
Yes	-	1910 (10)	2067 (11)
Do not know	-	465 (2)	478 (3)
**Suicide attempt**			
No	-	45 830 (96)	43 326 (95)
Yes	-	1528 (3)	1865 (4)
Do not know	-	500 (1)	571 (1)

^1^ Measured at birth. ^2^ Measured the year the adolescent turned 18 years. ^3^ Measured up until the adolescent turned 18 years. ^4^ Only available for participants replying to DNBC-18 after May 2019 (*N* = 19 186)

Lifetime prevalence of self-reported and hospital-recorded suicidality were calculated as the weighted percentage of participants who had experienced the assessed level of suicidality and presented with 95% confidence intervals (CI) using SAS and assuming a normal distribution. Analyses were stratified by sex and parental income. Parental income was categorised as yearly, total income level of both parents divided into quartiles and measured in the calendar year where adolescents turned 11 years i.e., before the typical onset of suicidality [1]. It was not possible to stratify analyses on both sex and parental income due to low numbers of hospital-recorded suicide attempt. Ratios between suicide attempts with and without hospital contact were calculated. In sub-analyses, the measures of suicidality were restricted to being within the year prior to participating in DNBC-18. In sensitivity analyses, we excluded those adolescents who responded ‘do not know’ from the analyses.

To evaluate validity of the applied algorithm, we calculated the proportion of participants with a hospital-recorded suicide attempt, who themselves reported lifetime suicide attempt or suicide ideation. These proportions are presented overall and according to the various definitions of hospital-recorded suicide attempt and by sex. In a sensitivity analysis, we restricted all measures of suicidality to the year prior to participating in DNBC-18. Proportions were presented with 95% CI calculated in SAS based on normal distribution.

## Results

Compared to the background population of all Danes born during the same period as DNBC children, the majority of the 47 858 included participants from DNBC-18 were girls. They were also more likely to have parents with higher income and educational level and in employment (Table 2). When applying the sample weights, participants resembled Danes born during the same period more with respect to the examined characteristics.

**Fig. 1:**
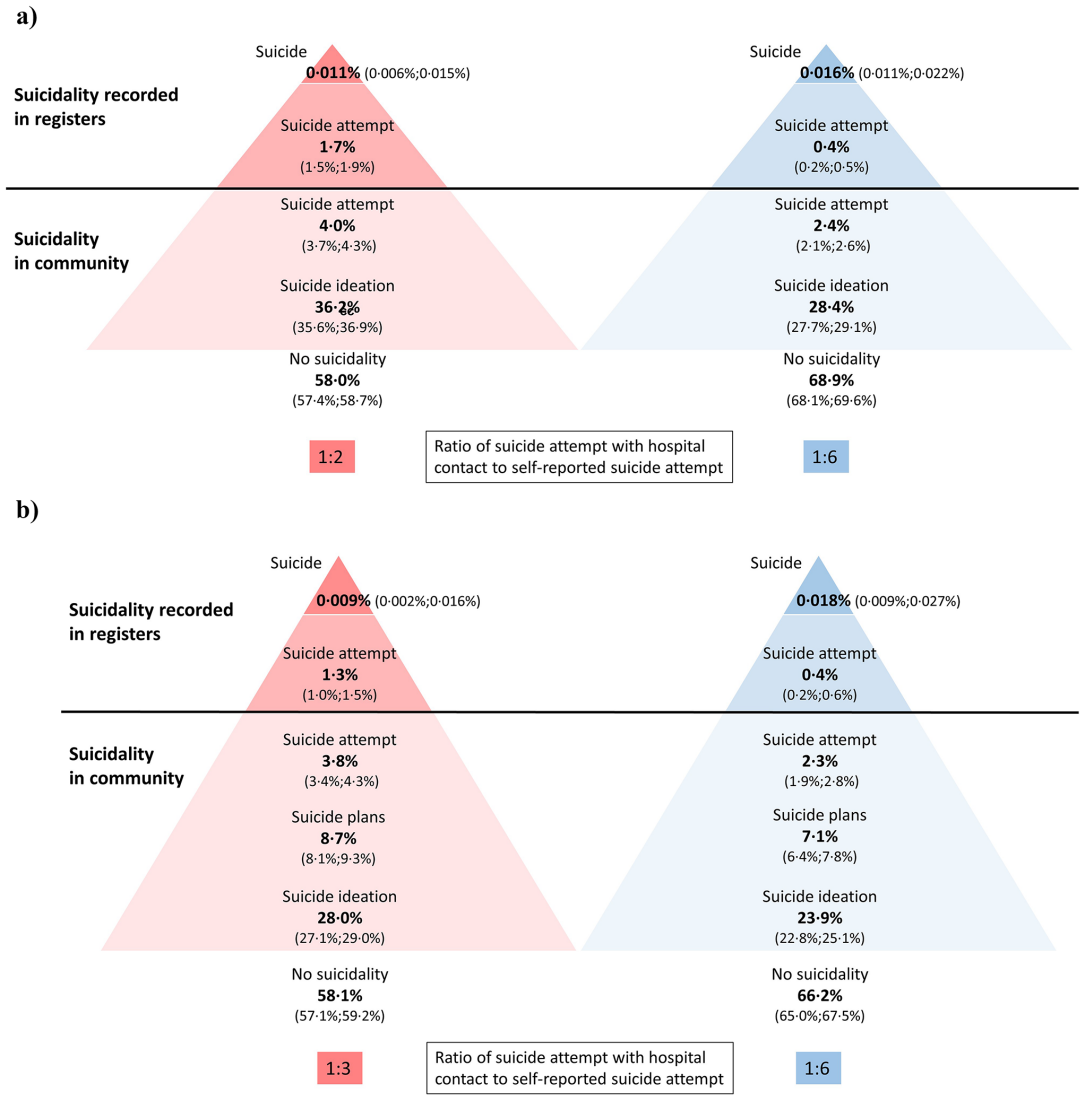
The prevalence of life-time suicidality (hierarchical) at age 18 among girls and boys with 95% confidence intervals and applied sample weights. **a**) Full sample. Girls (*N* = 27 727) Boys (*N* = 20 131). **b**) Sample with information on suicide plans. Girls (*N* = 11 277) Boys (*N* = 7909)

In the weighted sample of 18-year-old girls, 58·0% (95% CI 57·4%;58·7%) had never experienced any suicidality and 36·2% (95% CI 35·6%;36·9%) had experienced suicide ideation only (Fig. 1a). Among the remainder, 5·7% had had a suicide attempt: Of these, 4·0% (95% CI 3·7%;4·3%) had not been to hospital while 1·7% (95% CI 1·5%;1·9%) had a hospital-recorded suicide attempt. Among the 27 727 girls, 366 girls had a total of 570 hospital-recorded suicide attempts before age 18 years. In the weighted sample of boys, 68·9% (95% CI 68·1%;69·6%) had never experienced any suicidality, 28·4% (95% CI 27·7%;29·1%) had experienced suicide ideation only, and among the remainder, 2·7% had had a suicide attempt (Fig. 1a). Of these, 2·4% (95% CI 2·1%;2·6%) had not been to hospital, while 0·4% (95% CI 0·2%;0·5%) had a hospital-recorded suicide attempt. Among the 20 131 boys, 54 boys had a total of 65 hospital-recorded suicide attempts before age 18 years. Based on data for all Danes born during the same period as DNBC children, 23 girls and 38 boys corresponding to 0·011% (95% CI 0·006%;0·015%) and 0·016% (95% CI 0·011%;0·022%) respectively, had died by suicide before age 18 years. The ratio between hospital-recorded and self-reported suicide attempts was 1:2 for girls; implying that for every hospital presentation with a suicide attempt, two girls had had a suicide attempt and not been to hospital. The corresponding figure for boys was 1:6.

Of girls, 25·3% (95% CI 24·8%;25·9%) had experienced suicide ideation and 1·4% had a suicide attempt, either hospital-recorded (0·4%, 95 CI 0·3%;0·5%) or self-reported (1·0%, 95 CI 0·9%;1·2%), within the year prior to participating in DNBC-18 (Supplementary Table 4, Supplementary Fig. 2). Of boys, 19·0% (95% CI 18·4%;19·7%) had experienced suicide ideation and 0·7% had a suicide attempt, either hospital-recorded (0·1%, 95 CI 0·1%;0·2%) or self-reported (0·6%, 95 CI 0·5%;0·8%) within the year prior to participating in DNBC-18 (Supplementary Table 4, Supplementary Fig. 2).

In the sample including data on suicide plans (*N* = 19 186), 8·7% girls and 7·1% boys had had plans of suicide i.e., considered methods and made preparations (Fig. 1b).

Excluding participants replying ‘do not know’ to questions about suicidal ideation or behaviour did not alter the main results (Supplementary Table 5).

**Fig. 2:**
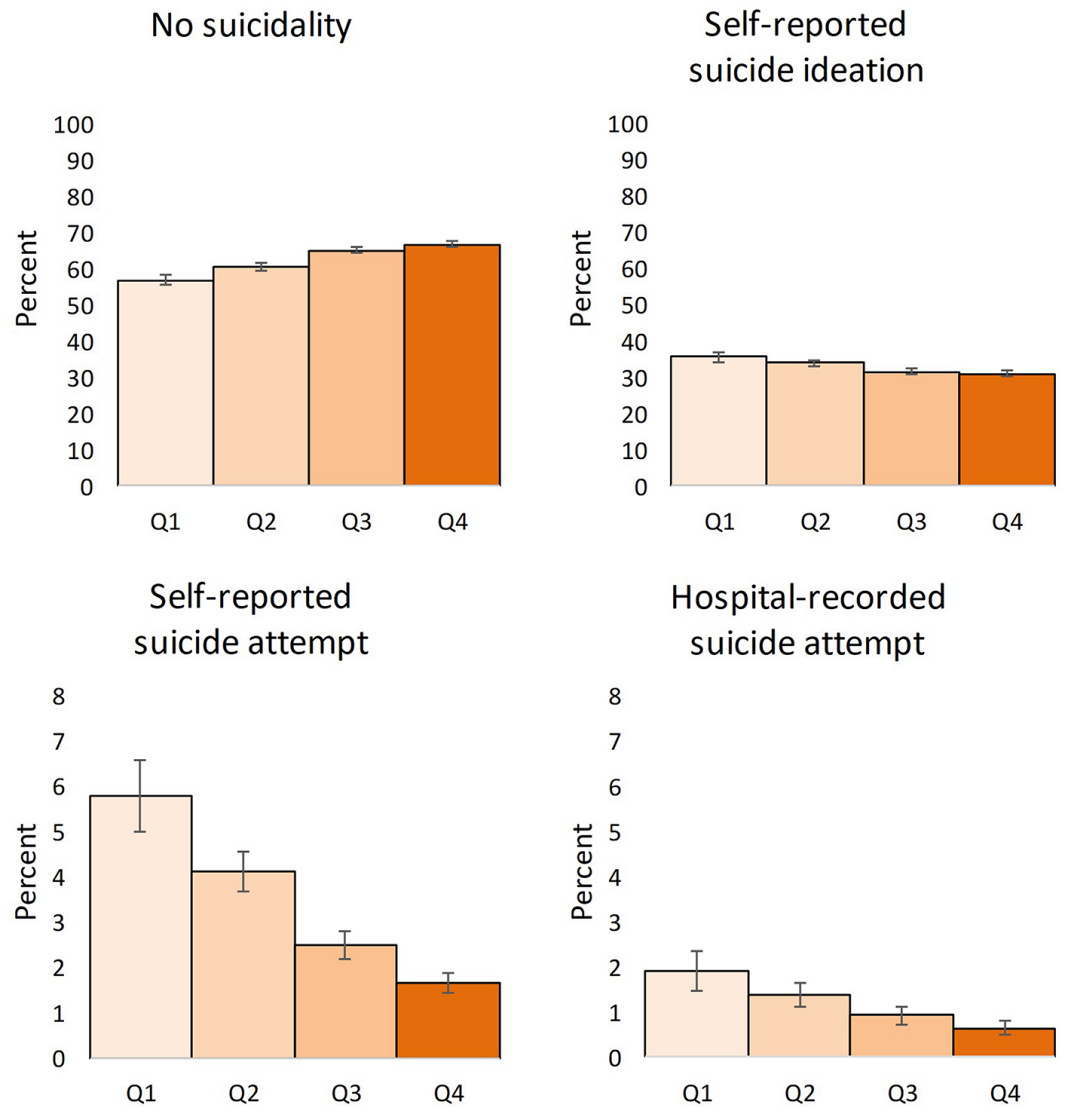
Weighted prevalence of life-time suicidality at age 18 in parental income quartiles at age 11 with 95% confidence intervals and applied sample weights Q1: Lowest income quartile (*N* = 6,174) Q2: Second lowest income quartile (*N* = 11,128) Q3: Second highest income quartile (*N* = 14,298) Q4: Highest income quartile (*N* = 16,215)

Little difference was observed across parental income groups for suicide ideation (Fig. 2). A higher prevalence of suicide attempts without (5·8%, CI 95% 5·0%;6·6%) and with hospital contact (1·9%, CI 95% 1·5%;2·3%) was found among participants with the lowest parental income, while the lowest prevalence of suicide attempts without (1·7%, CI 95% 1·4%;1·9%) and with hospital contact (0·7%, CI 95% 0·5%;0·8%) was found among those with highest parental income group. The ratio between suicide attempt with and without hospital contact was 1:3 across all income groups.

**Fig. 3:**
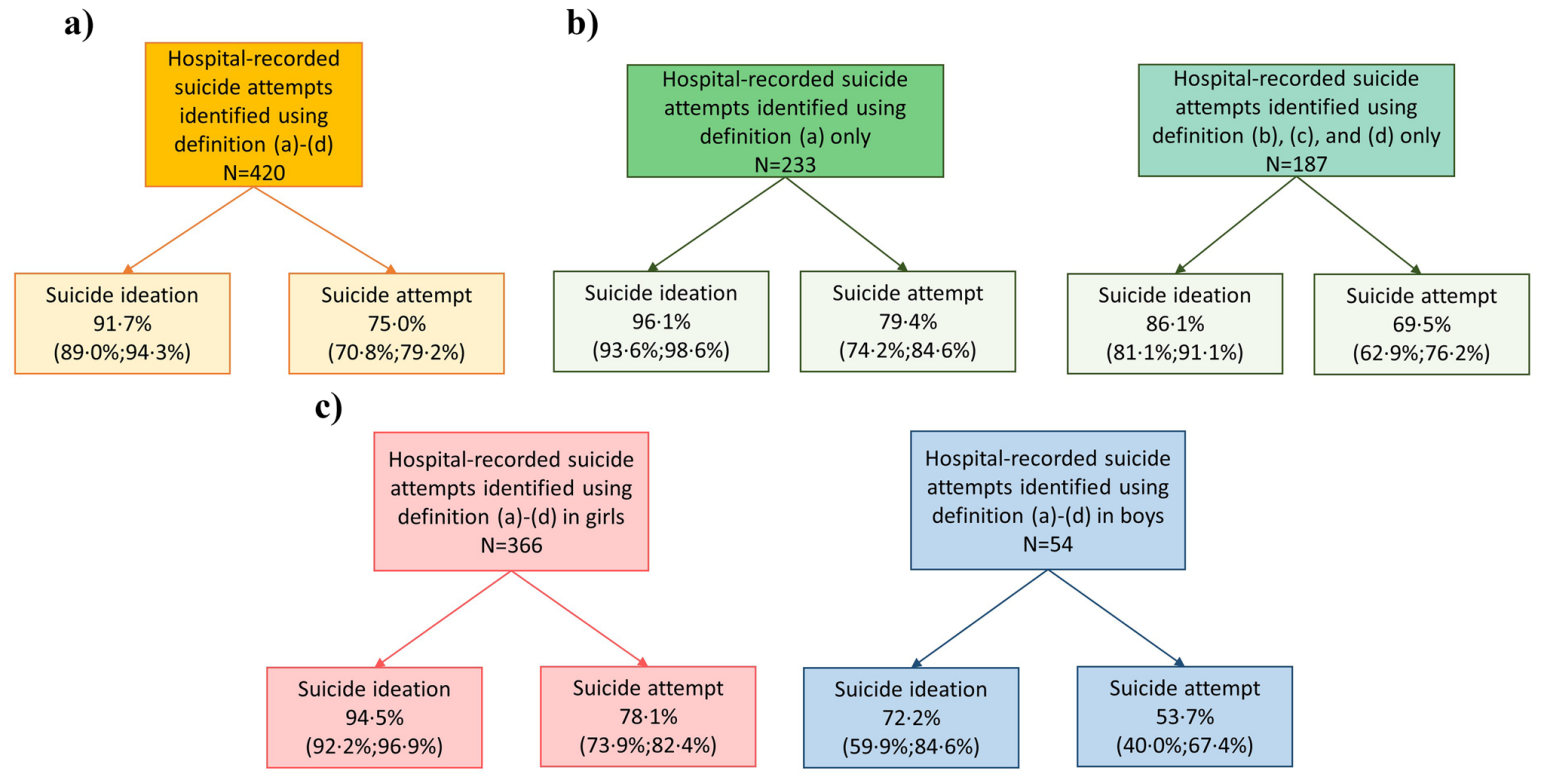
Assessment of the validity of hospital-recorded suicide attempt. **a**) Self-reported suicide ideation and suicide attempt^1^ in adolescents with hospital-recorded suicide attempt^2,3^**b**) Self-reported suicide ideation and suicide attempt^1^ in adolescents with a hospital-recorded suicide attempt identified by definition (a)^2^ and (b), (c), and (d)^3^ respetively **c**) Self-reported suicide ideation and suicide attempt^1^ in girls and boys with hospital-recorded suicide attempt^2,3 1^ Replied ‘yes’ to suicide ideation and/or suicide attempt. ^2^ (a) ICD-10 codes X60-X84 or where suicide attempt was recorded as the reason for contact (Table 1). ^3^ (b)-(d) A main diagnosis of accidental intoxication with weak analgesics or a main diagnosis of a psychiatric disorder in combination with a sub-diagnosis of either intoxication with specific drugs or injuries to the lower forearm (Table 1)

Among participants with a hospital-recorded suicide attempt, 91·7% (95% CI 89·0%;94·3%) self-reported having had suicide ideation and 75·0% (95% CI 70·8%;79·2%) self-reported having had suicide attempt (Fig. 3a). The alignment with self-reported suicidality was greatest when suicide attempt was specifically recorded in the record (Fig. 3b). For suicide attempts defined by hospital contacts that were likely to represent suicide attempts, the proportions of individuals with self-reported suicide ideation and suicide attempt were 86·1% (95% CI 81·1%;91·1%) and 69·5% (95% CI 62·9%;76·2) respectively (Fig. 3b). Girls with a hospital-recorded suicide attempt were more likely to self-report suicidality than boys (Fig. 3c). Among those with a hospital-recorded suicide attempt within the preceding year, 85·4% (95% CI 78·5%;92·4%) and 66·0% (95% CI 56·7%;75·3%) self-reported suicide ideation and suicide attempt within the last year, respectively (Supplementary Fig. 3).

## Discussion

In this study where self-reported information were linked with register data on an individual level, we found that approximately 1/3 of 18-year-olds had experienced thoughts of suicide, while 9% girls and 7% boys had more developed suicide thoughts in the form of planning by considering methods and making preparations. Additionally, almost 6% girls and 3% boys aged 18 years had had a suicide attempt. Our results further quantify the ‘hidden number’ of adolescents with suicide attempts, as for every girl and boy who presented to hospital with a suicide attempt, two girls and six boys, respectively, had reported a suicide attempt that had not resulted in hospital presentation. A social inequality was found for suicide attempts. The algorithm used to identify hospital-recorded suicide attempts aligned well with self-reported data, especially among girls.

A previous study indicated that among Danish high school students (the majority aged 15–20 years), 3·4% girls and 1·8% boys reported having ever attempted suicide [8]. This is lower than the estimates presented in this study, possibly because high school students represent a more well-functioning group than the DNBC-18 population that also includes adolescents who are not in high school. In an international meta-analysis, the life-time prevalence of suicide ideation, suicide plans and suicide attempt among children and young people aged 6–22 years was estimated to be 21%, 10% and 7%, respectively [11]. Thus, the prevalence of suicide ideation was higher in our study, while suicidal plans and attempts were less common.

Supporting previous research, we showed a substantial hidden number of adolescents with suicide attempts [5, 6]. Similar to our findings, a previous English study found the ‘hidden number’ to be higher in boys than girls in children aged 12–14 years although no sex-differences in the ratios were observed for adolescents aged 15–17 years [5]. In contrast, an Irish study found that the ‘hidden number’ was higher in girls than boys [6]. These diverging results could be explained by cultural and societal differences in treatment seeking and availabilities between countries and methodological differences between studies. Furthermore, the UK and Irish studies examined ‘self-harm’, which includes all intentional episodes of self-injury or self-poisoning irrespective of motive (including suicidal intent) [5], while in our study the adolescents were specifically asked whether they had tried to take their own life.

This is, to our knowledge, the first time that data on hospital- and self-reported suicide attempts, plans and ideation have been linked at an individual level. By integrating self-reported and hospital records for the same individuals, more exact estimates could be made. Additionally, previous literature was based on data from selected areas e.g., hospitals and cities, whereas this study used nationwide data and a well-defined background population of Danes born during the same period as participants with register data on suicide and hospital-recorded suicide attempts. Thereby we were able to calibrate findings to the background population of all children born in Denmark in 1996–2003.

Our findings support the perception that suicide attempts are more frequent in girls than boys [1, 5, 6]. However, a smaller sex-difference was found for self-reported suicide attempts compared to hospital-recorded suicide attempts, with a boy-to-girl ratio of 1:2 and 1:4 respectively. Thus, the sex-difference could be overestimated in studies where only hospital-recorded suicide attempts are included. Self-reported suicide ideation and suicide plans were likewise more common among girls, although the sex-difference was less substantial.

We demonstrated a social gradient, with the highest prevalence of suicide attempts among adolescents with low parental income. Danish residents have universal and free access to health care, which could explain the relatively constant ratio between suicide attempts with and without hospital contact across income groups. In countries with unequal access to health care, the ratio might vary more between income groups.

A part of the DNBC-18 data collection was conducted during the national lockdowns implemented to mitigate the COVID-19 pandemic. As we previously found the prevalence of suicidality in DNBC-18 to be similar before and during lockdown, we consider data collected in these periods to be comparable [22]. Even though healthcare-seeking at hospitals in general might have been compromised during lockdown, a Danish study comparing rates of hospital-recorded suicide attempt before and during the first and second lockdown did not observe this in adolescents [23]. 

The Danish algorithm used to identify hospital-recorded suicide attempt has previously been evaluated in all age-groups by researchers with a medical background reviewing medical records of 357 randomly identified cases of suicide attempt for descriptions of suicidal intentions [13]. The algorithm could reliably identify suicide attempts with suicidal intention in 42% men and 59% women. In our study among adolescents, we likewise found that the algorithm was more valid among girls. However, it is not possible to distinguish whether the algorithm is more likely to misclassify boys or boys are less likely to self-report suicide attempt. In the algorithm, suicidal intent is a prerequisite in one out of four definitions. The importance of suicidal intent and the distinction between non-suicidal self-injury and suicidal acts has been widely discussed [24, 25]. A psychiatric diagnosis in combination with injuries to the lower forearm or intoxication may reflect non-suicidal self-injury, misuse of drugs, or accidents rather than a suicide attempt. However, among individuals identified with a hospital recorded suicide attempt, without any confirmation of intent, i.e., by definition (b), (c), or (d), 86% reports having had suicidal ideation and 70% reported a suicide attempt. Despite a somewhat lower threshold than for records with a confirmed suicide attempt, this supports that the major share of people identified through definitions without confirmed intent did have a suicide attempt. The true number of suicide attempts with hospital presentation is expectedly in-between the number of suicide attempts identified by definition (a) and definitions (b), (c), and (d). The algorithm developed to identify suicide attempts in Danish register data can, thus, reliably be used for suicide research in Denmark, although it will not identify the large number of suicide attempts without hospital contact.

### Clinical implications

In average in every classroom of 30 pupils as many as 2–3 individuals aged 18 years have either seriously considered or attempted to take their own life, with girls and adolescent from lower income families being overrepresented. The high prevalence of suicide ideation among adolescents is especially concerning when taking the moderate to high transition rate to suicide attempt into consideration [26]. The substantial ‘hidden number’ of adolescents with suicide attempts is also concerning. Medical treatment following a suicide attempt is an opportunity for healthcare professionals to assess the underlying cause(s), which contributed to an individual’s suicidal behaviour, and refer them to appropriate treatment. Since many adolescents, especially boys, do not present to hospital following a suicide attempt or receive psychiatric treatment afterwards [27–29], offering help to individuals in the community is crucial for preventing repetition and addressing underlying mental health issues. Future research should address the mechanisms of community occurring suicide attempts and how these differ from attempts recorded at the hospital. A review has suggested that one out of three adolescents do not seek help after a suicide attempt, not even from informal sources, such as family members [7]. Secrecy could imply that appropriate help or treatment needed is not provided. This emphasizes the importance of ensuring that adolescents and their parents, who often serve as the first line of care takers, have knowledge and easy access to help, such as mental health hotlines and Suicide Prevention Clinics. The ‘hidden number’ of suicidality further underscores the need for community-based strategies for suicide prevention, preferably at an early age, as suicide ideation and behaviour may occur in childhood [30]. Several trials of school-based suicide prevention programs have yielded encouraging findings in reducing suicidality in school pupils [31]. Thus, a way forward could be implementing school-based suicide prevention as an important tool for early identification and intervention to prevent the development of suicidality [32]. This is particularly important among boys as they less commonly present to the hospital, especially considering that boys are more likely to die by suicide [2, 4]. 

### Strengths and limitations

Strengths of this study include the individual-level linkage of register-based and self-reported data, multiple measures of suicidality, a large well defined study population and utilization of sample weights. Pilot-testing ensured that questions on suicidality were interpreted as intended. Data on suicides from the Cause of Death register have previously been evaluated as reliable [33]. 

A limitation of this study is the possible underestimation of suicidality due to the fact that DNBC-18 constitute a selected population. Psychiatric studies have been suggested as particularly sensitive to attrition [34, 35]; however, when compared to individuals born in Denmark in the same years as the DNBC participants, the proportion having a psychiatric diagnosis was not markedly lower (11% vs. 12%). When sample weights were applied, the prevalence of suicidality increased, although this might still be somewhat underestimated. Hospital-recorded suicide attempts are under-recorded and the algorithm used to identify suicide attempts in the registers lacks both specificity and sensitivity; [12, 13] thus, implying a potential source of bias. Suicidality questions were not from a validated questionnaire and answers may be subject to reporting bias. The question about suicide ideation was vaguely phrased, with the formulation, ”*even though you would not do it,”* which might explain the very high frequency. It was not possible to estimate the extent of suicidality including suicide plans for the whole population, as data only was available for a sub-group. Information about contact with general practitioners regarding suicide ideations and attempts was not available but would have been valuable to this study. Given that information on frequency or timing (i.e., date) of the self-reported suicide attempt was not available, we could only estimate the proportion of the population with suicidality, whereas most research report rates of suicidality, i.e., based on more detailed information on time at risk. Further, adolescents with hospital-recorded suicide attempt may also have had other ‘hidden’ suicide attempts that did not lead to hospital contact.

In conclusion, a large proportion of adolescents experience suicidal ideation or behaviour, especially girls and adolescents in lower income families. In a classroom of 30 students, on average, 2–3 individuals will have either planned to or attempted to take their own life. Further, the substantial ‘hidden number’ indicate that the majority of adolescents do not seek hospital treatment immediately after a suicide attempt. Universal prevention strategies targeting adolescents with suicidality should be focused on the community, especially schools, and take account of social inequality.

## Electronic supplementary material

Below is the link to the electronic supplementary material.

Supplementary Material 1

## Data Availability

According to European law (General Data Protection Regulation), data containing potentially identifying or sensitive personal information are restricted. However, for academic research, data can be available subsequent to approval. Any request for DNBC data needs to follow the outlined procedures, see: https://www.dnbc.dk/access-to-dnbc-data. For access to the underlying person-level data in Danish registers, researchers need to apply to the Danish Health Data Authority and Statistics Denmark. The programming codes used on DNBC and register data in this study are available by contacting the corresponding author.
