# Supplementary material for: The iceberg model of suicidal ideation and behaviour in Danish adolescents: integration of national registry and self-reported data within a national birth cohort
Source: Eur Child Adolesc Psychiatry. 2024 Jun 25;34(2):611–21. doi: 10.1007/s00787-024-02503-w (PMC11868210; doi:10.1007/s00787-024-02503-w)
Supplement: Supplementary file 1 — Supplementary Material 1 [file 787_2024_2503_MOESM1_ESM.docx]

**Supplementary**

**Supplementary Table 1**. Data sources for register-based variables included in the analyses.

| **Variable** | **Register** |
| --- | --- |
| Maternal age at birth | Danish Medical Birth Register^1^ |
| Parity |  |
| Parental income | Income Statistics Register^2^ |
| Parental job status | The Employment Classification Module^3^ |
| Highest parental education | Danish Education Registers^4^ |
| Out-of-home placement | The Register of Support for Children and adolescents^5^ |
| Sex | Danish Civil Register^6^ |
| Co-living parents |  |
| Psychiatric diagnosis in adolescent | The Danish National Patient Register^7^ and the Psychiatric Central Research Register^8^ |
| Parental psychiatric diagnosis |  |
| Hospital recorded suicide attempt |  |
| Suicide | Cause of Death Register^9^ |
| ^1^Bliddal M, Broe A, Pottegård A, Olsen J, Langhoff-Roos J. The Danish Medical Birth Register. *Eur J Epidemiol* 2018; **33**: 27–36 ^2^Baadsgaard M, Quitzau J. Danish registers on personal income and transfer payments. *Scand J Public Health* 2011; **39**: 103–5. ^3^Petersson F, Baadsgaard M, Thygesen LC. Danish registers on personal labour market affiliation. *Scand J Public Health* 2011; **39**: 95–8. ^4^Jensen VM, Rasmussen AW. Danish education registers. *Scand J Public Health* 2011; **39**: 91–4. ^5^ https://www.dst.dk/da/Statistik/dokumentation/statistikdokumentation/anbringelser-af-boern-og-unge/indhold ^6^Pedersen CB. The Danish Civil Registration System. *Scand J Public Health* 2011; **39**: 22–5. ^7^Lynge E, Sandegaard JL, Rebolj M. The Danish National Patient Register. *Scand J Public Health* 2011; **39**: 30–3. ^8^Mors O, Perto GP, Mortensen PB. The Danish Psychiatric Central Research Register. *Scand J Public Health* 2011; **39**: 54–7. ^9^Helweg-Larsen K. The Danish Register of Causes of Death. *Scand J Public Health* 2011; **39**: 26–9 | |

**Supplementary Table 2.** Weighted and unweighted prevalence (with 95% CI) of suicidality at age 18 by sex and parental income.

|  |  | **Self-reported** | | **Register based** | |
| --- | --- | --- | --- | --- | --- |
|  | No suicidality | Suicide ideation | Suicide attempt | Suicide attempt | Suicide^1^ |
| **Total**  N=47,858 | | | | | |
| **Weighted^2^** | 63·4%  (62·9%;63·9%) | 32·4%  (31·8%;32·8%) | 3·25%  (3·04%;3·46%) | 1·1%  (1·0%;1·2%) | N/A |
| **Unweighted** | 64·7% (64·3%;65·1%) | 31·9% (31·5%;32·3%) | 2·5% (2·4%;2·7%) | 0·9%  (0·8%;1·00%) | 0·014%  (0·010%;0·017%) |
| **Sex** N=47 858 | | | | | |
| **Weighted^3^** | | | | | |
| Girls  **(**N=27 727) | 58·0%  (57·4%;58·7%) | 36·2%  (35·6%;36·9%) | 4·0%  (3·7%;4·3%) | 1·7%  (1·5%;1·9%) | N/A |
| Boys  (N=20 131) | 68·9%  (68·1%;69·6%) | 28·41%  (27.69%;29·13%) | 2·4%  (2·1%;2·6%) | 0·4%  (0·3%;0·5%) | N/A |
| **Unweighted** | | | | | |
| Girls  **(**N=27 727) | 60·4%  (59·8%;61·0%) | 35·2%  (34·7%;35·8%) | 3·1%  (2·9%;3·3%) | 1·3%  (1·2%;1·4%) | 0·011%  (0·006%;0·015%) |
| Boys  (N=20 131) | 70·7%  (70·0%;71·3%) | 27·3%  (26·7%;27·9%) | 1·8%  (1·6%;2·0%) | 0·3%  (0·2%;0·3%) | 0·016%  (0·011%;0·022%) |
| **Parental income** N=47,815 | | | | | |
| **Weighted^4^** | | | | | |
| Q1   (N=6174) | 56·8%  (55·3%;58·3%) | 35·5%  (34·1%;36·9%) | 5·8%  (5·0%;6·6%) | 1·9%  (1·5%;2·3%) | N/A |
| Q2   (N=11 128) | 60·6%  (59·6%;61·6%) | 33·9%  (33·0%;34·9%) | 4·1%  (3·7%;4·5%) | 1·4%  (1·1%;1·7%) | N/A |
| Q3  (N=14 298) | 65·1%  (64·3%;65·9%) | 31·5%  (30·8%;32·3%) | 2·5%  (2·2%;2·8%) | 0·9%  (0·7%;1·1%) | N/A |
| Q4  (N=16 215) | 66·6%  (65·9%;67·4%) | 31·1%  (30·3%;31·8%) | 1·7%  (1·4%;1·9%) | 0·7%  (0·5%;0·8%) | N/A |
| **Unweighted** | | | | | |
| Q1   (N=6174) | 58·3%  (57·1%;59·5%) | 35·6%  (34·4%;36·8%) | 4·5%  (4·0%;5·0%) | 1·6%  (1·3%;1·9%) | 0·016%  (0·009%;0·024%) |
| Q2   (N=11 128) | 62·0%  (61·1%;62·9%) | 33·4%  (32·6%;34·3%) | 3·5%  (3·1%;3·8) | 1·1%  (0·9%;1·3%) | 0·012%  (0·005%;0·018%) |
| Q3  (N=14 298) | 66·5%  (65·7%;67·2%) | 30·6%  (29·8%;31·3%) | 2·2%  (2·0%;2·4%) | 0·8%  (0·6%;0·9%) | 0·017%  (0·009%;0·025%) |
| Q4  (N=16 215) | 67·5%  (66·7%;68·2%) | 30·5%  (29·8%;31·2%) | 1·5%  (1·3%;1·7%) | 0·6%  (0·5%;0·7%) | 0·010%  (0·004%;0·016%) |
| ^1^ Suicides were based on the population of people born in Denmark from mid 1996 - mid 2003 for whom there were complete information on sex (N=451,768) and parental income (N=446,517).  ^2^ Weights were calculated based on sex, maternal age at birth, and parity (measured at birth) and parental income, highest parental education, co-living parents, out-of-home placement, any childhood and adolescent psychiatric diagnosis, and any history of parental psychiatric diagnosis (measured at age 18).  ^3^ Weights were calculated based on maternal age at birth, and parity (measured at birth) and parental income, highest parental education, co-living parents, out-of-home placement, any childhood and adolescent psychiatric diagnosis, and any history of parental psychiatric diagnosis (measured at age 18).  ^4^ Weights were calculated based on sex, maternal age at birth, and parity (measured at birth) and highest parental education, co-living parents, out-of-home placement, any childhood and adolescent psychiatric diagnosis, and any history of parental psychiatric diagnosis (measured at age 18). | | | | | |

**Supplementary Table 3.** Weighted and unweighted prevalence of suicidality at age 18 by sex in sub-population including data on suicide plans presented with 95% CI.

|  |  | **Self-reported** | | | **Register based** | | |
| --- | --- | --- | --- | --- | --- | --- | --- |
|  | No suicidality | Suicide ideation | Suicide plans | Suicide attempt | Suicide attempt | Suicide^1^ |  |
| **Total**  N=19 186 | | | | | | | |
| **Weighted^2^** | 62·0%  (61·2%;62·8%)  63·9%  (63·2%;64·6%) | 26·0%  (25·2%;26·7%  25·6%  (25·0%;26·3%) | 8·0%  (7·5%;8·4%)  7·3%  (6·9%;7·6%) | 3·2%  (2·9%;3·5%)  2·5%  (2·3%;2·7%) | 0·9%  (0·7%;1·1%)  0·8%  (0·6%;0·9%) | N/A |  |
| **Unweighted** |  |  |  |  |  | 0·014%  (0·008%;0·020%) |  |
| **Sex**  N=19 186 | | | | | | | |
| **Weighted^3^** | | | | | | | |
| Girls  (N=11 277) | 58·1% (57·1%;59·2%)  66·2% (65·0%;67·5%) | 28·0% (27·1%;30·0%)  23·9% (22·8%;25·1%) | 8·7% (8·1%;9·3%)  7·1% (6·4%;7·8%) | 3·8%  (3·4%;4·3%)  2·3%  (1·9%;2·8%) | 1·3%  (1·0%;1·5%)  0·4%  (0·2%;0·6%) | N/A  N/A |  |
| Boys  (N=7 909) |  |  |  |  |  |  |  |
| **Unweighted** |  |  |  |  |  |  |  |
| Girls  (N=11 277)  Boys  (N=7 909) | 60·5% (59·6%;61·4%)  68·7% (67·7%;69·8%) | 27·5% (26·7%;28·3%)  23·0% (22·1%;23·9%) | 8·0% (7·5%;8·5%)  6·2% (5·7%;6·8%) | 3·0%  (2·7%;3·3%)  1·7%  (1·5%;2·0%) | 1·1% (0·88%;1·26%)  0·3%  (0·2%;0·4%) | 0·009%  (0·002%;0·016%)  0·018%  (0·009%;0·027%) |  |
| ^1^Suicides are based on the population of people born in Denmark from February 2001-June 2003 (N=153,233)  ^2^Weights based on sex, maternal age at birth, and parity (measured at birth) and parental income, highest parental education, co-living parents, out-of-home placement, any childhood and adolescent psychiatric diagnosis, and any history of parental psychiatric diagnosis (measured at age 18)  ^3^Weights based on maternal age at birth, and parity (measured at birth) and parental income, highest parental education, co-living parents, out-of-home placement, any childhood and adolescent psychiatric diagnosis, and any history of parental psychiatric diagnosis (measured at age 18) | | | | | | | |

**Supplementary Table 4.** Sensitivity analyses of prevalence of suicidality within the year prior to participating in DNBC-18 by sex and presented with 95% CI.

|  |  | **Self-reported** | | **Register based** |
| --- | --- | --- | --- | --- |
|  | No suicidality | Suicide ideation | Suicide attempt | Suicide attempt |
| **Sex**  N=47 858 | | | | |
| **Weighted^1^** | | | | |
| Girls  **(**N=27 727) | 73·3%  (72·7%;73·9%) | 25·3%  (24·8%;25·9%) | 1·0%  (0·9%;1·2%) | 0·4%  (0·3%;0·5%) |
| Boys (N=20 131) | 80·3%  (79·6%;80·9%) | 19·0%  (18·4%;19·7%) | 0·6%  (0·5%;0·8%) | 0·1%  (0·1%;0·3%) |
| **Unweighted** | | | | |
| Girls  **(**N=27 727) | 75·0%  (74·5%;75·5%) | 24·0%  (23·4%;24·6%) | 0·7%  (0·6%;0·8%) | 0·3%  (0·2%;0·4%) |
| Boys (N=20 131) | 81·6%  (81·1%;82·2%) | 17·9%  (17·3%;18·4%) | 0·4%  (0·3%;0·5%) | 0·1%  (0·0%;0·1%) |
| ^1^Weights based on maternal age at birth, and parity (measured at birth) and parental income, highest parental education, co-living parents, out-of-home placement, any childhood and adolescent psychiatric diagnosis, and any history of parental psychiatric diagnosis (measured at age 18) | | | | |

**Supplementary Table 5.** Sensitivity analyses where those who answered ‘do not know‘ to questions regarding suicidality were excluded.

|  |  | **Self-reported** | | **Register based** |
| --- | --- | --- | --- | --- |
|  | No suicidality | Suicide ideation | Suicide attempt | Suicide attempt |
| **Sex**  N=45 284 | | | | |
| **Weighted^1^** | | | | |
| Girls  (N=26 067) | 56·6%  (56·0%;57·3%) | 37·4%  (36·7%;38·1%) | 4·2%  (3·9%;4·5%) | 1·7%  (1·5%;1·9%) |
| Boys  (N=19 217) | 68·0%  (67·2%;68·8%) | 29·2%  (28·4%;29·9%) | 2·5%  (2·2%;2·7%) | 0·4%  (0·2%;0·5%) |
| **Unweighted** | | | | |
| Girls  (N=26 067) | 58·9%  (58·3%;59·5%) | 36·5%  (35·9%;37·1%) | 3·3%  (3·1%;3·5%) | 1·3%  (1·2%;1·4%) |
| Boys  (N=19 217) | 69·8%  (69·2%;70·5%) | 28·0%  (27·4%;28·7%) | 1·9%  (1·7%;2·1%) | 0·3%  (0·2%;0·3%) |
| ^1^Weights based on maternal age at birth, and parity (measured at birth) and parental income, highest parental education, co-living parents, out-of-home placement, any childhood and adolescent psychiatric diagnosis, and any history of parental psychiatric diagnosis (measured at age 18) | | | | |

**Supplementary Figure 1.** Flowchart of the study population and background population


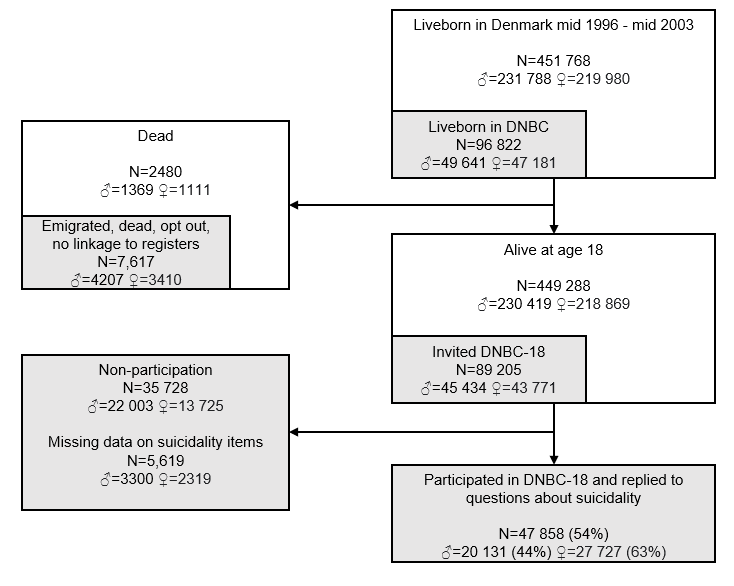


**Supplementary Figure 2.** The prevalence of self-reported suicide ideation and -attempts and hospital-recorded suicide attempts (hierarchical) within the year prior to participating in DNBC-18 (N=47 858) among girls and boys with 95% confidence and applied sample weights.


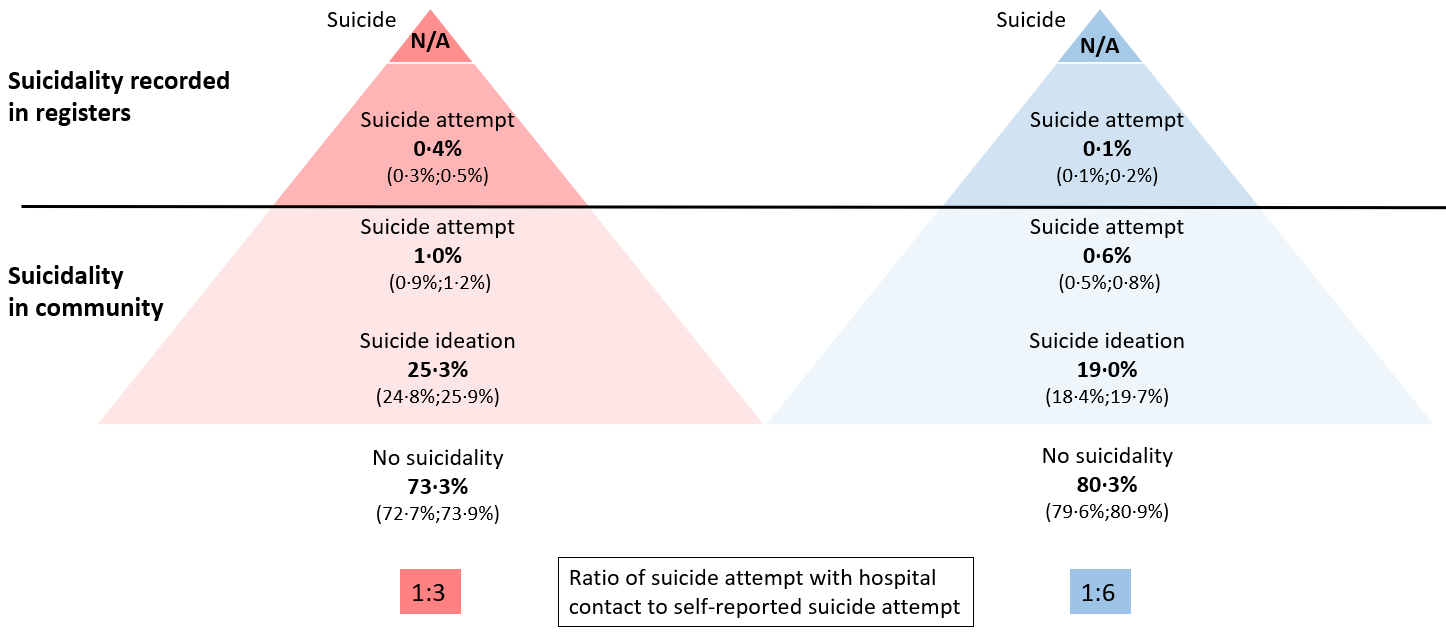


**Supplementary Figure 3.** Assessment of the validity of hospital-recorded suicide attempt. Self-reported suicide ideation and suicide attempt within the last year^1^ in adolescents with hospital-recorded suicide attempt within last year before participating in DNBC-18^2,3^


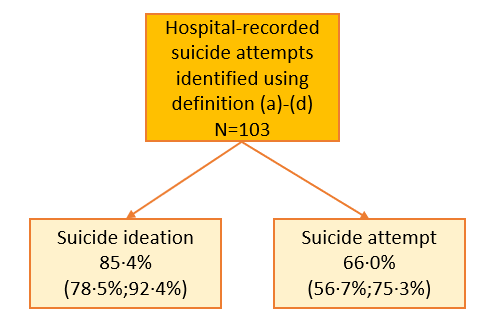


^1^ Replied ‘yes’ to suicide ideation and/or suicide attempt within the last year.
^2^ (a) ICD-10 codes X60-X84 or where suicide attempt was recorded as the reason for contact (Table 1).
^3^ (b)-(d) A main diagnosis of accidental intoxication with weak analgesics or a main diagnosis of a psychiatric disorder in combination with a sub-diagnosis of either intoxication with specific drugs or injuries to the lower forearm (Table 1).
